# An increase in the posterior subarachnoid space accelerates the timing of syrinx resolution after foramen magnum decompression of type I Chiari malformation

**DOI:** 10.1038/s41598-021-98546-z

**Published:** 2021-09-27

**Authors:** Yuichiro Ohnishi, Sho Fujiwara, Tomofumi Takenaka, Saki Kawamoto, Koichi Iwatsuki, Haruhiko Kishima

**Affiliations:** 1 Department of Neurosurgery, Graduate School of Medicine, Osaka University, 2-2 Yamadaoka, Suita, Osaka Japan; 2 Department of Neurosurgery, Osaka Gyoumeikan Hospital, Osaka, Japan

**Keywords:** Spinal cord diseases, Headache, Spinal cord diseases

## Abstract

Syrinx resolution has been associated with an increase in the size of the posterior subarachnoid space (pSAS) after foramen magnum decompression (FMD) for type I Chiari malformation (CM1). The present study investigated the influence of pSAS increase on syrinx resolution and symptom improvement after FMD. 32 patients with CM1 with syrinx were analyzed retrospectively. FMD was performed for the 24 patients with CM1 with syrinx. pSAS areas were measured on sagittal magnetic resonance images. Neurological symptoms were grouped into three clinical categories and scored. The rates of symptom improvement in the CM1 patients with syrinx after FMD was 19.7% ± 12.9%. The mean times to the improvement of neurological symptoms in CM1 patients with syrinx after FMD was 23.4 ± 50.2 months. There were no significant differences between the patients with and without improvement of syrinx after FMD with regard to the age, length of tonsillar herniation, BMI, and preoperative pSAS areas. The rate of increase in the pSAS areas was significantly higher in the group with syrinx improvement within 1 year (p < 0.0001). All patients with a > 50% rate of increase in the pSAS area showed syrinx improvement. Our results suggested that the increasing postoperative pSAS area accelerated the timing of syrinx resolution.

## Introduction

Type I Chiari malformation (CM1) is an anatomical abnormality characterized by cerebellar tonsillar herniation through the foramen magnum^1,2^. In CM1 patients, the mean position is 13 mm below the foramen magnum with a range from 3 mm below the foramen magnum to 29 mm below^3^. In general, 5 mm of caudal descent of the tonsil is considered a CM1. Symptoms are thought to originate from an impaired dynamic cerebrospinal fluid (CSF) flow^2,4^ and direct compression of the brain stem^5,6^.

Syringomyelia in CM1 patients has risk of subsequent spinal cord damage. Symptoms of syringomyelia include muscle weakness, loss of reflexes, loss of sensitivity to pain and temperature, headaches, stiffness in your back, shoulders, arms and legs, pain in your neck, arms and back, and scoliosis. The cause of syrinx formation is not yet fully understood. However, previous studies have shown that patients with a smaller posterior fossa volume are more likely to demonstrate abnormalities of the CSF flow at the foramen magnum and have an increased risk of developing syrinx than others^7–13^.

The aim of surgery is to relieve central nervous system compression and improve CSF circulation. Although syrinx resolution has been associated with an increase in the posterior subarachnoid space (pSAS) volume after surgery^14^, the significance of the extent of this volume increase on syrinx resolution remains unclear. In this study, we retrospectively investigated the effect of the extent of pSAS increase on syrinx resolution and symptom improvement after foramen magnum decompression (FMD) in CM1 patients.

## Methods

All methods were carried out in accordance with relevant guidelines and regulations.

### Patient population

32 patients with CM1 with syrinx (ages, 36.1 ± 15.9 years old; male 7, female 25) were analyzed retrospectively. FMD was performed for 24 patients with CM1 with syrinx. Duraplasty was done for four patients. The timing of surgery was determined by a shared decision-making process involving both physicians and patients. Surgery was recommended for patients who experienced debilitating symptoms. Patients with mild symptoms who requested to reduce neurological symptoms were also treated surgically. Debilitating and mild symptoms indicate Score 1/2 and Score 3/4 in Neurological scores, respectively (Table 1). All human studies were approved by the ethical review board of Osaka University Medical Hospital (No. 17098). All patients provided informed consent prior to inclusion in this study.

**Table 1:** Neurological scores.

Score	Pain	Sensory disturbances	Motor disturbances
5	None	Normal	Full power
4	Slight, no medication	Present, not significant	Movement against resistance
3	Good control with medication	Significant, function not restricted	Movement against gravity
2	Insufficient control with medication	Some restriction of function	Movement without gravity
1	Severe despite medication	Severe restriction of function	Contraction without movement

Symptoms were grouped into three clinical categories: pain, sensory disturbance, and motor disturbance.

Table 1 shows the characteristics of the FMD patients with syrinx. We divided the FMD patients with syrinx according to the time of syringomyelia improvement: those who showed improvement within the first postoperative year were classified as having early improvement, and those whose condition improved later were classified as having late improvement. We also divided the FMD patients with syrinx according to the timing of symptom improvement: those who showed improvement within the first postoperative year were classified as having early improvement, and those who improved later were classified as having late improvement.

### Radiological assessments

All patients underwent magnetic resonance imaging (MRI) of the cervicothoracic spine and brain. All patients had one or more syrinxes on preoperative MRI. The diagnosis of CM1 was made based on MRI findings and defined as tonsillar herniation at least 5 mm below the level of the foramen magnum. Follow-up MRI was performed every 6 to 12 months.

**Figure 1:**
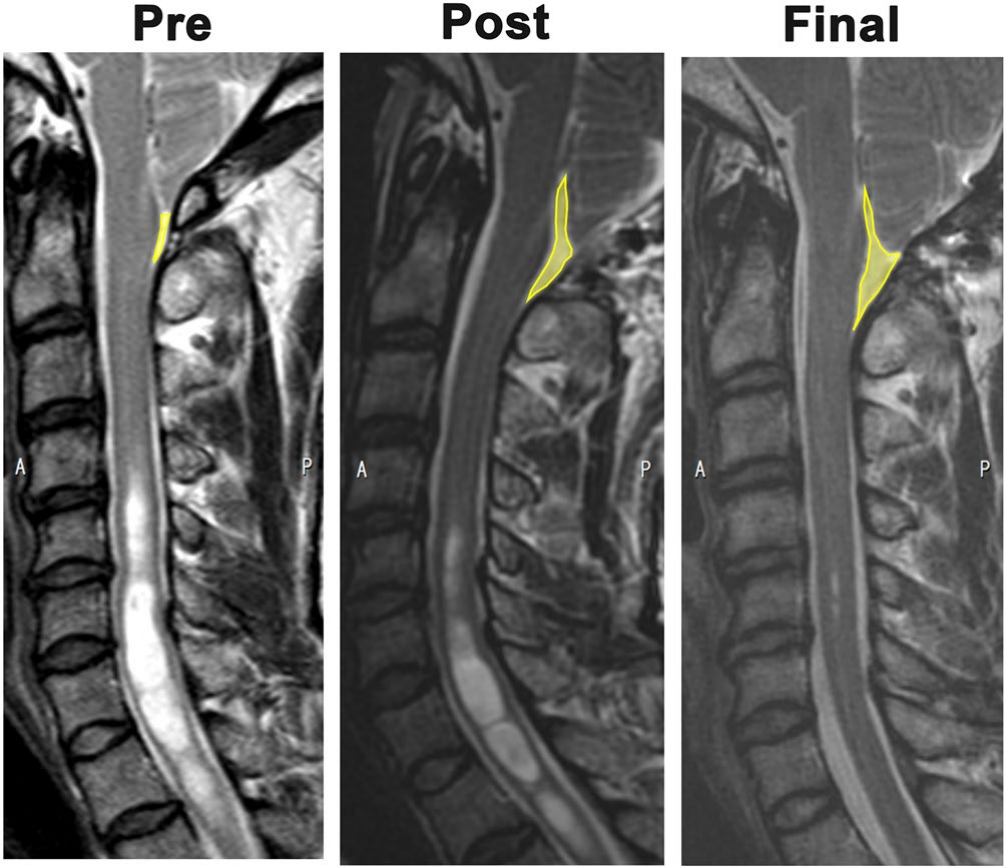
pSAS was defined as the area between the dura mater and spinal cord from the foramen magnum to C2 on a midline sagittal image on T2-weighted imaging. The yellow line represents the outline of the pSAS. Representative MRI image with syrinx improvement. Right, preoperative images; Middle, postoperative images; Left, images at final follow-up.

We measured the area of the pSAS between the cerebellar tonsils and C2 in the midline sagittal image on T2-weighted imaging (T2WI) before surgery, immediately after surgery, and at follow-up. The sagittal slice position and orientation were adjusted so that the slice plane was perpendicular to the cervical vertebral body in anterior–posterior axis and left–right axis. The mid-sagittal slice was selected from multiplanar (axial, coronal, sagittal) images. The pSAS area was obtained using manual delineation with the Image J software program^15^. Two examiners measured the pSAS area. We calculated the inter-rater reliability for the pSAS measurements. ICC2Ck was 0.995. pSAS was enlarged sequentially over time after FMD (Fig. 1). The ratios of the pSAS area immediately after surgery and at follow-up to the preoperative pSAS area were calculated. The percentage increase in pSAS area was calculated by dividing the difference between the pre- and postoperative values by the preoperative value. Syrinx resolution was defined as a decreased syrinx area on midline sagittal images of T2WI. The maximum syrinx/spinal cord length ratio and overall syrinx length were also measured to assess syrinx resolution after FMD. A 20% reduction in the syrinx/cord ratio or overall length at the last follow-up was defined as syrinx resolution.

### Neurological assessments

Symptoms and clinical examination results were obtained from the medical records. A neurological score was given to each patient according to a previously described scoring system (Table 1)^16^. Symptoms were grouped into three clinical categories: pain, sensory disturbance, and motor disturbance. Clinical data were collected at the initial, perioperative, and follow-up examinations. The percentage increase in the improvement of the neurological score was calculated by dividing the difference between the pre- and postoperative values by the preoperative value.

### Surgical procedure

A midline skin incision was made extending from just below the inion to the spinous process of the C2 vertebra. We performed exposure of the occipital bone, including defining the borders of the foramen magnum. We then dissected the posterior arch of the C1 vertebra in a subperiosteal fashion. Suboccipital craniectomy with C1 laminectomy was performed with a high speed drill or craniotome and rongeurs in all patients to achieve wide decompression of the cerebellar hemispheres, midline structures, brainstem, and spinal cord. The width and height of the bony opening was 25 to 30 mm. For posterior fossa decompression without duraplasty (PFD), the atlanto-occipital ligament was divided, and the underlying outer dura leaflet was incised and resected. For posterior fossa decompression with duraplasty (PFDD), opening dura was performed in a caudal to rostral fashion. Cranial dura has 2 layers. Dural sinuses lie between these 2 layers. There were no venous structures at the level of the C1. Dural opening proceeded rostrally, maintaining hemostasis by applying bipolar electrocautery or by Weck clips or suture on each side. Arachnoid opening was performed sharply. Using Weck clips or suture to affix the arachnoid to the cut dural edge. Care was taken to avoid contamination of the subarachnoid space with blood. Y-shaped duraplasty was performed with a Gore-Tex® dura substitute (W.L. Gore & Associates Inc., Flagstaff, AZ, USA). The water tight closure was performed with fibulin glue in all patients to guard against potential CSF leakage not noted intraoperatively. No patient developed meningitis, CSF leakage, or wound infection.

### Statistical analyses

All statistical analyses were performed using the XLSTAT software program (Addinsoft Inc., NY, USA). Wilcoxon’s signed-rank test was used to compare continuous variables between two groups. For comparisons among multiple groups, data were analyzed using the Kruskal–Wallis test along with Dunn's post hoc test. The values were presented as the mean ± standard deviation.

### Disclosure

The authors report no conflicts of interest concerning the materials or methods used in this study or the findings specified in this paper.

### IRB approval

Ethical review board approval was obtained.

## Results

32 patients with CM1 with syrinx was (ages, 36.1 ± 15.9 years old; male 7, female 25) were analyzed retrospectively. The length of tonsillar herniation and preoperative pSAS areas in the CM1 patients with syrinx was 10.5 ± 4.3 (mm) and 81.2 ± 76.2 (mm^2^), respectively. FMD was performed for 24 patients with CM1 with syrinx (ages, 33.6 ± 16.2 years old; male 5, female 19). The length of tonsillar herniation and preoperative pSAS areas in the FMD patients was 11.2 ± 4.2 (mm) and 49.2 ± 35.8 (mm^2^), respectively. The rates of symptom improvement in the CM1 patients with syrinx after FMD was 19.7% ± 12.9%. The mean times to the improvement of neurological symptoms in CM1 patients with syrinx after FMD was 23.4 ± 50.2 months. The rates of increase in the pSAS areas in CM1 patients with syrinx after FMD was 169.6% ± 383.4%.

Table 2 shows the characteristics in the CM1 patients with syrinx after FMD. Among CM1 patients with syrinx after FMD, 18 showed improvement in syrinx, while 6 did not. Four cases underwent PFDD. The ages of the patients with and without improvement of syrinx after FMD were 35.1 ± 13.5 years and 29.1 ± 23.5 years, respectively (p = 0.525). The lengths of tonsillar herniation in patients and without improvement of syrinx after FMD were 11.0 ± 4.2 mm, and 11.8 ± 4.6 mm, respectively (p = 0.733). The BMI in patients with and without improvement of syrinx after FMD were 23.7 ± 4.3, and 19.7 ± 3.9, respectively (p = 0.071). The preoperative pSAS areas in patients with and without improvement of syrinx after FMD were 40.1 ± 18.4 mm^2^, and 52.2 ± 40.0 mm^2^, respectively (p = 0.626). There were no significant differences between the patients with and without improvement of syrinx after FMD with regard to the age, length of tonsillar herniation, BMI, and preoperative pSAS areas.

**Table 2:** Characteristics of the CM1 patients with syrinx after FMD.

	Total	Syrinx improvement (–) after FMD	Syrinx improvement ( +) after FMD	P value
Number of patients	24	6	18	N.D
Age (years)	33.6 ± 16.2	35.1 ± 13.5	29.1 ± 23.5	0.525
**Sex**				
Male	5	0	5	N.D
Female	19	6	13	N.D
Tonsillar herniation (mm)	11.2 ± 4.2	11.0 ± 4.2	11.8 ± 4.6	0.733
BMI	22.7 ± 4.6	19.7 ± 3.9	23.7 ± 4.3	0.071
Number of PFD/PFDD	20/4	6/0	14/4	N.D
Preoperative pSAS areas (mm^2^)	49.2 ± 35.8	40.1 ± 18.4	52.2 ± 40.0	0.626

N.D., not determined.

**Figure 2:**
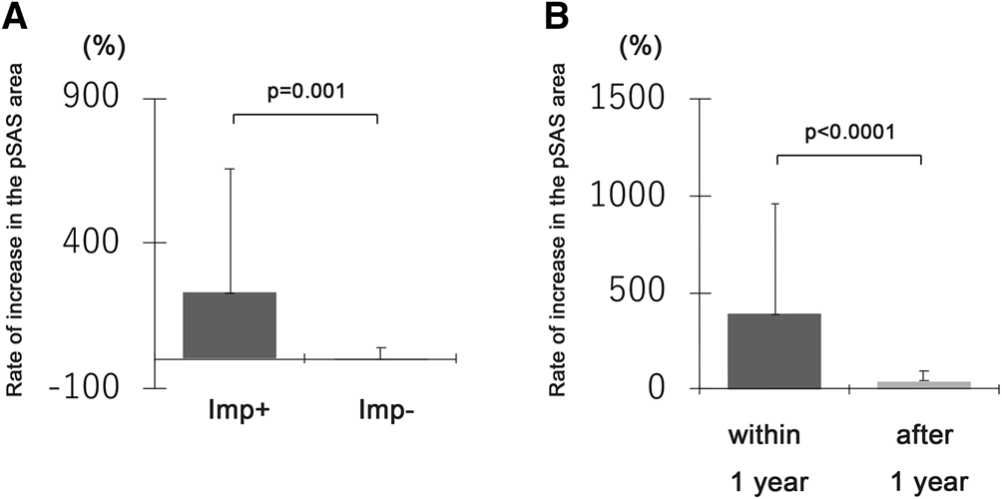
The rate of increase in the pSAS area after FMD. (**A**) CM1 patients in the group with syrinx improvement was significantly higher rate of increase in the pSAS areas after FMD. (**B**) the rate of increase in the pSAS areas after FMD was significantly higher in the group with syrinx improvement within 1 year compared with the group with improvement after 1 year. Imp + : syrinx improvement positive, Imp-: syrinx improvement negative.

**Figure 3:**
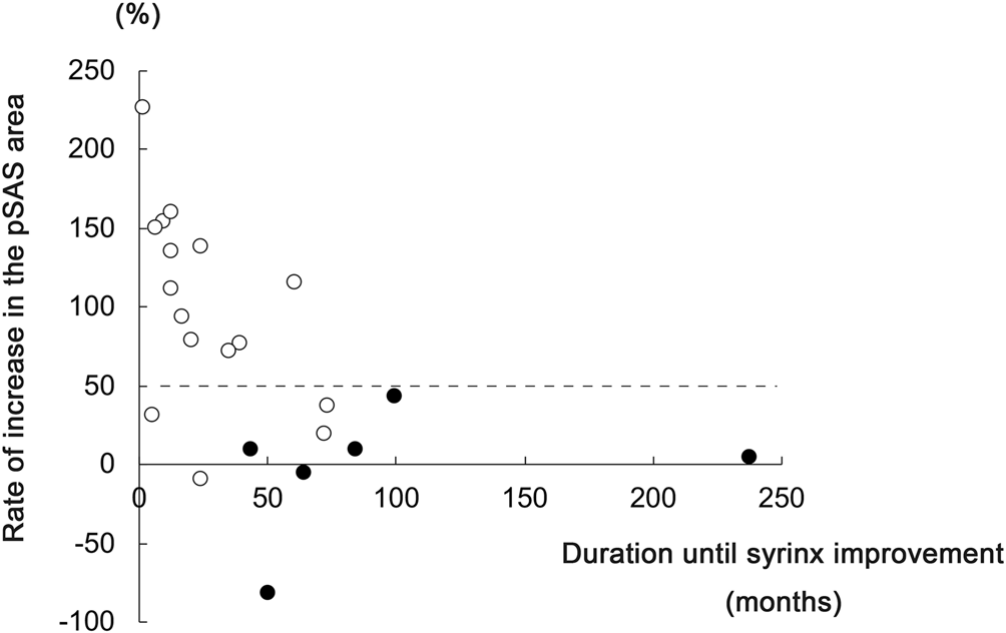
Scatter plot showing the time to syrinx resolution. The vertical axis is the rate of increase in the pSAS area after FMD. The horizontal axis is the duration until syrinx improvement. Points over 250% on the vertical axis were not presented. White and black circles indicate syrinx improvement and no improvement, respectively. The dashed line indicates a 50% rate of increase in the pSAS area.

In CM1 patients with syrinx, the mean time to the improvement of syrinx was 42.1 ± 50.4 months after FMD. The pre- and postoperative pSAS areas were 49.2 ± 35.9 and 95.3 ± 76.7 (mm^2^), respectively. CM1 patients in the group with syrinx improvement was significantly higher rate of increase in the pSAS areas after FMD (Fig. 2A). Furthermore, the rate of increase in the pSAS areas after FMD was also significantly higher in the group with syrinx improvement within 1 year compared with the group with improvement after 1 year (Fig. 2B). Although there was no correlation between the rate of increase in the pSAS areas after FMD and the timing of syrinx resolution (R^2^ = 0.081), all patients with a > 50% rate of increase in the pSAS area after FMD showed syrinx improvement (Fig. 3). However, syrinx improvement was limited to 40% among patients with a < 50% rate of increase in the pSAS area. These findings suggest that the increasing postoperative pSAS area accelerated the timing of syrinx resolution in CM1 patients with syrinx.

## Discussion

FMD re-establishes the CSF flow pathway and resolves syringomyelia^15^. Patients with a crowded posterior fossa are more likely to experience syrinx formation than those without obvious posterior fossa crowdedness^17^. Sahuquillo et. al stated that enlargement of the cisterna magna is critical to clinical improvement^18^. In the present study, CM1 patients with improvement of syrinx showed a marked rate of increase in the pSAS area after FMD. All patients a > 50% rate of increase in the pSAS area after FMD showed syrinx improvement. Thus, syrinx resolution can be expected in cases with a postoperative pSAS area > 1.5 times the preoperative pSAS area. Furthermore, the rate of increase in the pSAS area after FMD was significantly higher in the group with symptom improvement within 1 year than in the group with improvement after 1 year. These findings suggest that the increase in the postoperative pSAS area accelerated the timing of syrinx resolution.

In CM1 patients with syrinx, the pre-operative pain, sensory disturbance, and motor disturbance scores were 3.0 ± 1.1, 3.4 ± 1.2, and 4.6 ± 0.6, respectively, and the respective postoperative scores were 4.0 ± 0.8, 4.2 ± 0.8, and 4.8 ± 0.3. The postoperative scores significantly improved for pain and sensory disturbance (p = 0.0019 and 0.0059, respectively) but not motor disturbance (p = 0.1636). The pre- and postoperative neurological scores was 11.1 ± 1.6 and 13.1 ± 1.3, respectively (p = 0.00002). There was no correlation between the rate of increase in the symptoms after FMD and the timing of improvement in the symptoms after FMD (R^2^ = 0.311) or between the rate of increase in the pSAS areas after FMD and the rate of increase in the symptoms after FMD (R^2^ = 0.049).

FMD patients improved symptoms and syrinx^19,20^. There is debate regarding the risks and benefits of PFD versus PFDD. Meta-analysis shows no convincing evidence that one method is superior over the other^21^. Another meta-analysis presented that PFD is a safe and effective surgical procedure with comparable outcomes and fewer complications compared to PFDD^22^. However, it has been reported that PFDD can be an optimal surgical strategy because of its higher clinical improvement and lower recurrence rate in the patients with syringomyelia^23^. Furthermore, good symptom control has been achieved despite radiographic failure^24^. Therefore, there is little to choose between PFD and PFDD, establishing post-surgical morphometric standards may lead to select the effective and safe surgical procedure.

Our study is limited by its retrospective design and reliance on imaging interpretation, which inherently lends itself to internal bias. In addition, we analyzed only single sagittal slices; the evaluation of multiple slices may have been more accurate. Other limitations include the selection bias inherent to noncontrolled surgical studies.

## Conclusions

Our results suggest that the increase in the postoperative pSAS area accelerated the timing of syrinx resolution However, the study is limited by its retrospective and observational design. Further investigations are needed to better understand how best to treat CM1 in patients with syringomyelia.
